# Peer-led team learning in an undergraduate biology course: Impacts on recruitment, retention, and imposter phenomenon

**DOI:** 10.1186/s13104-023-06338-7

**Published:** 2023-05-09

**Authors:** Mariah C. Maxwell, Julia J. Snyder, Ryan D. P. Dunk, Jeremy D. Sloane, Isabella Cannon, Jason R. Wiles

**Affiliations:** 1 Syracuse University, Syracuse, NY USA; 2 Auburn University, Auburn, AL USA; 3 Skidmore College, Saratoga Springs, NY USA

**Keywords:** Peer-led team learning (PLTL), Recruitment, Retention, Imposter phenomenon, Women, Underrepresented minority (URM), First-generation college student, Biology, Undergraduate, Gateway course

## Abstract

**Objectives:**

The data presented in this note were collected during a multi-year project conducted in the context of large-enrollment introductory biology course at a large private R-1 research institution in the Northeastern United States. The project aimed to examine the impact of Peer-Led Team Learning (PLTL) on the recruitment and retention of marginalized groups in Science, Technology, Engineering, and Mathematics (STEM) majors. While several results from the project have been published, additional data of interest have yet to be reported. This data note reports on additional associations between PLTL participation and improved outcomes for students from groups that have historically been excluded in STEM. Additional data reported herein were collected to determine if students in the course experienced imposter phenomenon, and whether PLTL may be associated with reduced levels of imposter feelings.

**Data description:**

The data in this note includes academic information such as final course grades and academic level; socio-demographic information such as gender identity, minority status, and first-generation status; and information on student recruitment, retention, imposter feelings, and participation in Peer-Led Team Learning (PLTL). These data might be useful and of value to education researchers and undergraduate STEM instructors who are interested in improving equity in STEM education.

## Objective

For over a decade, major professional organizations have called for reform in traditional Science, Technology, Engineering, and Mathematics (STEM) education by using more active learning strategies [1]. These calls have been in response to a large and growing body of evidence affirming that active learning is more effective and equitable than traditional lecture [2, 3].

Peer-Led Team Learning (PLTL) is a well-studied active learning model wherein students meet in small groups to collaboratively solve problem sets related to course content [4]. These groups are led by a peer leader who is trained in facilitating teamwork, discussion, and problem solving. Participating in PLTL can improve students’ academic achievement and retention [5], especially for students who have historically been marginalized in STEM [6, 7].

PLTL may improve student retention by mitigating the impostor phenomenon, which describes individuals with internal feelings that they lack talent and skill despite significant accomplishments and achievements [8]. Imposter feelings could result in attrition of well-qualified students from STEM fields.

The data in this note were collected during a large project centered on the same introductory biology course. The objective for data collection associated with Data File 1 (Data File 1) was to measure the potential impact of PLTL on the recruitment and retention of students from marginalized populations in STEM. Some results from this study were published by Sloane and colleagues [6], but Data file 1 includes additional information that may be of interest. The objectives for collection of data contained in Data File 4 (Data File 4) were to determine the degree to which students in the course may experience imposter phenomenon, and to determine if students may be less likely to struggle with imposter feelings through exposure to and interactions with potential role models (peer leaders).

Detailed methods of PLTL implementation and data collection are described along with additional descriptions of the study population in [6, 7, 9].

## Data description

Both data files in this note were collected within the context of an introductory biology course at a large, private, research-intensive institution in the Northeastern United States. With regard to gender, no students reported identifying beyond the binary.

### Data files 1–3

The data collection methods for the data in Data file 1 can be found in the corresponding publication [6], along with results describing the impact of PLTL on the recruitment and retention of underrepresented minoritized (URM) students in STEM majors. Chi-square analyses were used to examine whether first-generation college students and women who participated in PLTL were more likely to be retained in STEM majors than their counterparts who did not.

Retention By First-Generation Status (Data file 2).

Among students who did not engage in PLTL, no difference in STEM retention rates was observed between first-generation and continuing generation students (*X*^2^ = 0.340, N = 101, df = 1, p = .560). Continuing generation students who engaged in PLTL were retained in STEM majors at a higher rate than their counterparts who did not engage in PLTL, although this difference is not significant at an alpha level of 0.05 (*X*^2^ = 2.575, N = 127, df = 1, p = .109). First-generation students who engaged in PLTL were significantly more likely to be retained in STEM majors than first-generation students who did not engage in PLTL (*X*^2^ = 3.969, N = 34, df = 1, p = .046).

Retention by Gender (Data file 3).

Among students who did not engage in PLTL, women were significantly less likely to be retained in STEM majors than men (*X*^2^ = 4.998, N = 101, df = 1, p = .026). Men who engaged in PLTL were retained in STEM majors at a higher rate than their counterparts who did not engage in PLTL, although this difference is not significant at an alpha level of 0.05 (*X*^2^ = 0.883, N = 57, df = 1, p = .346). Women who engaged in PLTL were significantly more likely to be retained in STEM majors than women who did not participate in PLTL (*X*^2^ = 6.066, N = 104, df = 1, p = .014). Among the students who engaged in PLTL, no significant differences in the retention of men and women (*X*^2^ = 0.684, N = 60, df = 1, p = .408).

### Data files 4–6

The information in Data file 4 was collected at the end of one semester of introductory biology. Imposter feelings were measured using Clance Imposter Phenomenon Scale (CIPS), which consists of 20 items measuring the extent to which participants experience imposter feelings [8]. Participants responded on a 5-point Likert scale ranging from 1 (not at all true) to 5 (very true). The CIPS is scored by adding together the numbers of the responses to each statement, with higher scores indicating greater levels of imposter feelings. Additional student data that were collected include final course grade, gender, ethnicity, and participation in PLTL.

A main-effects general linear model was used to analyze the impacts of gender, year in school, major, race/ethnicity, course grade, and participation in PLTL on imposter score. Estimated marginal means were extracted from the model and compared between genders.

When accounting for the impact of other variables in the model, both gender (*F*_1,336_ = 8.68, p < .01) and the number of PLTL sessions attended (*F*_1,336_ = 4.1021, p < .05) had a significant impact on imposter scores (Data file 5). The more PLTL sessions students attended, the lower their imposter scores tended to be. Estimated marginal means showed that when accounting for other variables in the model, men had an average impostor score of 57.4 ± 2.0 and women had an average impostor score of 61.9 ± 2.3 (Data file 6).

**Table 1:** Overview of data files/data sets

Label	Name of data file/data set	File types (file extension)	Data repository and identifier (DOI or accession number)
Data file 1	Recruitment and Retention Data	.xlsx	10.3886/E174421V4-125301 [10]
Data file 2	Retention by First Generation Status	.jpg	10.3886/E174421V4-125302 [11]
Data file 3	Retention by Gender	.jpg	10.3886/E174421V4-125303 [12]
Data file 4	Imposter Phenomenon Data	.xlsx	10.3886/E174421V4-125304 [13]
Data file 5	GLM Model of Students’ Imposter Scores	.xlsx	10.3886/E174421V4-125305 [14]
Data file 6	Imposter Score by Gender	.jpg	10.3886/E174421V3-124679 [15]

## Limitations

- When examining self-reported data one must keep in mind that respondents may vary in how they ascribe values to their feelings. Self-reported data may also be subject to several types of biases, such as social desirability bias or recall bias. Additionally, self-reported data may vary with how individual participants feel during the time that they take the survey.
- The data presented in this data note are the result of natural experiments, in that students were not randomly assigned to control (non-PLTL) and experimental (PLTL) groups.

## Data Availability

The data described in this Data note can be freely and openly accessed on Open Inter-University Consortium for Political and Social Research (openICPSR) under 10.3886/E174421V3. Please see Table 1 and references [10–15] for details and links to the data.

## Abbreviations

CIPS: Clance imposter Phenomenon Scale
HHMI: Howard Hughes Medical Institute
OIR: Office of Institutional Research
PLTL: Peer-Led Team Learning
STEM: Science, Technology, Engineering, and Mathematics
URM: Underrepresented Minoritized* *Note: URM has historically denoted populations whose representation in STEM fields is lower than the proportion of these groups among the general population. Characterizing these groups as “underrepresented minorities” is a statistical term that ignores the exclusion that has led to underrepresentation. In order to recognize that the minority status of these groups in STEM fields is a result of exclusionary practices, when using URM, we recognize that the underrepresentation of these groups is due to their having been “minoritized”. A better term, which we hope to see normalized in the field, is Persons Excluded due to Ethnicity or Race (PEER) [16, 17].

## References

[1] American Association for the Advancement of Science (AAAS). Science without borders. Washington, D.C.; 2011.

[2] Freeman S, Eddy S, McDonough M, Smith M, Okoroafor N, Jordt H (2014). Active learning increases student performance in science, engineering, and mathematics. Proc Natl Acad Sci.

[3] Theobald EJ, Hill MJ, Tran E, Agrawal S, Nicole Arroyo E, Behling S (2020). Active learning narrows achievement gaps for underrepresented students in undergraduate science, technology, engineering, and math. Proc Natl Acad Sci.

[4] Gosser D, Roth Vi, Gafney L, Kampmeier J, Strozak V, Varma-Nelson P et al. Workshop chemistry: Overcoming the barriers to student success. Chem Educ. 1996;1(1).

[5] Wilson SB, Varma-Nelson P (2016). Small groups, significant impact: a review of peer-led Team Learning research with implications for STEM education researchers and faculty. J Chem Educ.

[6] Sloane JD, Dunk RDP, Snyder JJ, Winterton CI, Kelly M, Wiles JR. Peer-Led Team Learning is associated with an increased retention rate for STEM majors from marginalized groups. Proc NABT Res Symp. 2021. https://nabt.org/files/galleries/Sloane_et_al_Wiles_NABT_Symposium_2021.pdf.

[7] Snyder JJ, Sloane JD, Dunk RDP, Wiles JR (2016). Peer-led Team Learning helps minority students succeed. PLoS Biol.

[8] Clance PR, Imes SA (1978). The imposter phenomenon in high achieving women: Dynamics and therapeutic intervention. Psychother Theory Res Pract.

[9] Snyder JJ, Wiles JR. Peer-led team learning in introductory biology: Effects on peer leader critical thinking skills. PLoS ONE. 2015;10(1). 10.1371/journal.pone.0115084. 10.1371/journal.pone.0115084 PMC4309528 25629311

[10] Maxwell MC, Snyder JJ, Dunk RDP, Sloane JD, Cannon I, Wiles JR (2022). Peer-led team learning in an undergraduate biology course: impacts on recruitment, retention, and imposter phenomenon. OpenICPSR.

[11] Maxwell MC, Snyder JJ, Dunk RDP, Sloane JD, Cannon I, Wiles JR (2022). Peer-led team learning in an undergraduate biology course: impacts on recruitment, retention, and imposter phenomenon. OpenICPSR.

[12] Maxwell MC, Snyder JJ, Dunk RDP, Sloane JD, Cannon I, Wiles JR (2022). Peer-led team learning in an undergraduate biology course: impacts on recruitment, retention, and imposter phenomenon. OpenICPSR.

[13] Maxwell MC, Snyder JJ, Dunk RDP, Sloane JD, Cannon I, Wiles JR (2022). Peer-led team learning in an undergraduate biology course: impacts on recruitment, retention, and imposter phenomenon. OpenICPSR.

[14] Maxwell MC, Snyder JJ, Dunk RDP, Sloane JD, Cannon I, Wiles JR (2022). Peer-led team learning in an undergraduate biology course: impacts on recruitment, retention, and imposter phenomenon. OpenICPSR.

[15] Maxwell MC, Snyder JJ, Dunk RDP, Sloane JD, Cannon I, Wiles JR (2022). Peer-led team learning in an undergraduate biology course: impacts on recruitment, retention, and imposter phenomenon. OpenICPSR.

[16] Asai D, Excluded. JMBE. 2020;21(1). 10.1128/jmbe.v21i1.2071 PMC7148151 32313599

[17] Asai D. Race Matters. 2020;181(4). 10.1016/j.cell.2020.03.044 32413295

